# The Systemic Risk Factors for the Development of Infectious Keratitis after Penetrating Keratoplasty: A Population-Based Cohort Study

**DOI:** 10.3390/diagnostics14182013

**Published:** 2024-09-11

**Authors:** Yung-Nan Hsu, Whei-Ling Chiang, Jing-Yang Huang, Chia-Yi Lee, Shih-Chi Su, Shun-Fa Yang

**Affiliations:** 1 Institute of Medicine, Chung Shan Medical University, Taichung 402, Taiwan; 2 Department of Physical Therapy and Rehabilitation, Chang Bing Show Chwan Memorial Hospital, Changhua 505, Taiwan; 3 School of Medical Laboratory and Biotechnology, Chung Shan Medical University, Taichung 402, Taiwan; 4 Center for Health Data Science, Chung Shan Medical University Hospital, Taichung 402, Taiwan; 5 Nobel Eye Institute, Taipei 115, Taiwan; 6 Whole-Genome Research Core Laboratory of Human Diseases, Chang Gung Memorial Hospital, Keelung 204, Taiwan; 7 Department of Medical Biotechnology and Laboratory Science, College of Medicine, Chang Gung University, Taoyuan 333, Taiwan; 8 Department of Medical Research, Chung Shan Medical University Hospital, Taichung 402, Taiwan

**Keywords:** infectious keratitis, penetrating keratoplasty, diabetes mellitus, chronic ischemic heart disease, epidemiology

## Abstract

Penetrating keratoplasty (PK) is a corneal surgery that is employed to repair the full-thickness corneal lesion. This study aimed to survey the possible systemic risk factors of infectious keratitis after penetrating keratoplasty (PK) via the Taiwan National Health Insurance Research Database (NHIRD). A retrospective case–control study was conducted, and 327 patients who received the PK were enrolled after exclusion. The main outcome was the development of infectious keratitis, and people were divided into those with infectious keratitis and those without the outcome. Cox proportional hazard regression was conducted to produce adjusted hazard ratios (aHRs) and 95% confidence intervals (CIs) of specific demographic indexes and systemic diseases on infectious keratitis. There were 68 patients who developed infectious keratitis after the whole follow-up period. The diabetes mellitus (DM) (aHR: 1.440, 95% CI: 1.122–2.874, *p* = 0.0310) and chronic ischemic heart disease (aHR: 1.534, 95% CI: 1.259–3.464, *p* = 0.0273) groups demonstrated a significant association with infectious keratitis. The DM group also revealed significant influence on infectious keratitis development in all the subgroups (all *p* < 0.05). Nevertheless, the effect of chronic ischemic heart disease on infectious keratitis was only significant on those aged older than 60 years (*p* = 0.0094) and both sexes (both *p* < 0.05). In conclusion, the presence of DM and chronic ischemic heart disease are associated with infectious keratitis after PK. However, local risk factors for infectious keratitis developed in those receiving PK had not been evaluated.

## 1. Introduction

Penetrating keratoplasty (PK) is a corneal surgery that is usually used to manage the full-thickness corneal lesion [1]. Indications of PK including the keratoconus, pseudophakic/aphakic bullous keratopathy, and keratitis, which have regional differences [2]. The postoperative visual acuity of PK is acceptable, with more than half of individuals reaching 20/40 [3], but the refractive outcome is often unpredictable [4]. The common complications of PK are persistent corneal epithelial defect, stitches rupture, elevated intraocular pressure, and graft rejection [5,6].

Regarding the severe complications of PK, infectious keratitis could be one of the most dreadful conditions [6]. The infectious keratitis in PK is challenging to handle, and only 43% of patients experiencing post-PK infectious keratitis can maintain the corneal graft clarity [7]. In the advanced status of infectious keratitis, another PK may be warranted to eradicate the infectious tissue on the graft cornea [8]. Despite timely treatment, the visual outcome of infectious keratitis after PK cannot be promised, and severe visual impairment still exists frequently [9]. In recent studies, the presence of bacteria is the most common etiology of post-PK infectious keratitis [10], while the fungal infection after the PKP surgeries also accounts for more than 15% of cases with post-PK infectious keratitis [11].

Several risk factors had been proposed for the occurrence of infectious keratitis [12,13]. For instance, contact lens wear, pre-existing corneal abrasion, superficial keratitis, dry eye disease, eyelid disease, and trauma correlate with the development of infectious keratitis [12]. In a recent study with more than 1000 cases of post-PK infectious keratitis, the patients aged equal or more than 85 years showed a significantly higher risk of developing infectious keratitis [14]. Still, the middle-aged population still has a chance to develop post-PK infectious keratitis [15]. In addition, rejection risk and postoperative epithelial complications were independently associated with post-PK infectious keratitis [16], and diabetes mellitus (DM), neoplastic disorders, and atopy diseases had been proposed as possible risk factors for post-PK infectious keratitis [17]. Despite some previous research studies demonstrating the risk factor of post-PK infectious keratitis with adequate patient numbers [14,16], the systemic risk factor for infectious keratitis developed in those receiving PK had not been totally evaluated. Furthermore, some systemic diseases like DM would weaken the corneal condition and lead to corneal epithelial defects [18], and both DM and chronic ischemic heart disease associate with the infection in other sites/tissues; thus, they may have some effects on corneal infection [19,20]. Accordingly, the systemic disease may relate to infectious keratitis development after PK, which needs further evaluation.

The purpose of our study is to investigate the possible systemic risk factors of infectious keratitis in people who received PK via the operation of the National Health Insurance Research Database (NHIRD) of Taiwan. Also, the subgroup analyses stratified by age and sex were also employed.

## 2. Materials and Methods

### 2.1. Data Source

All the methods performed in our study adhered to the Declaration of Helsinki of 1964 and the late amendments. Our study was approved by both the Institutional Review Board of Chung Shan Medical University (project identification code: CS1-20108) and the National Health Insurance Administration of Taiwan. The need for written informed consent was abandoned by the above two institutions. The NHIRD of Taiwan stores the claimed data of the Taiwan health insurance system, in which approximately 23 million Taiwanese individuals were involved. The data interval of the NHIRD ranged from 1 January 2000 to 31 December 2016. Available information in the NHIRD involves the International Classification of Diseases, Ninth Revision (ICD-9) diagnostic code, the International Classification of Diseases, Tenth Revision (ICD-10) diagnostic code, age, sex, education level, medical department, inhabitant region, image intervention codes, laboratory intervention codes, surgery and procedure codes, and the international ATC codes for medicines. In our study, the longitudinal health insurance database (LHID) 2000 version, a sub-database derived from NHIRD, was consumed. The LHID 2000 contains information about two million Taiwanese derived from the NHIRD. Patients in the LHID 2000 were followed at same interval in the NHIRD.

### 2.2. Patient Selection

In our retrospective case–control study, individuals receiving PK were defined as follows: (1) receipt of PK according to the existence of surgery codes; (2) receipt of steroid and antibiotic therapy after the PK procedure according to related ATC codes; and (3) the PK procedure was performed by an ophthalmologist according to the department code. The index date was set as the date right after the PK procedure. To raise the homogeneity of our participants and erase some extreme disorders, the following exclusion criteria were adopted in our study: (1) participants younger than 20 years old or older than 100 years since we want to exclude those minors and extreme elderly, (2) blindness before the index date, (3) the diagnosis of an ocular tumor before the index date, (4) suffered from infectious endophthalmitis within 3 months before the index date, (5) suffered from dry eye disease within 3 months before the index date based on the related ICD-9/ICD-10 codes, (6) suffered from epithelial defect within 3 months before index date based on the related ICD-9/ICD-10 codes, and (7) infectious keratitis was observed before the index date. We excluded the dry eye disease and epithelial defect because we want to exclude the ocular surface factors for infectious keratitis and focus on the influence of systemic co-morbidity. After the exclusion, a total of 327 participants that received PK were enrolled in our analyses.

### 2.3. Main Outcome Definition

The main outcome in the current study was the infectious keratitis. In further detail, only the infectious keratitis that achieved the subsequent conditions was accounted for as outcome development in our study: (1) the presence of infectious keratitis-related ICD-9/ICD-10 diagnostic codes; (2) the performance of corneal scraping, smear exams, and culture exams based on procedure codes; (3) the application of oral or topical anti-microbial agents after the emergence of infectious keratitis diagnosis; and (4) the infectious keratitis diagnosis was made by an ophthalmologist. We tracked our patients until the presence of infectious keratitis, the individual withdrawn from the National Health Insurance, or till the end of NHIRD/LHID 2000, which indicates the 31st December 2016.

### 2.4. Systemic Co-Morbidity Enrollment

To evaluate the effects of demographic characteristics and systemic disorders in infectious keratitis development, the following parameters were collected according to the accompany codes in the LHID 2000: age, sex, urbanization level, hypertension, cerebrovascular disease, diabetes mellitus (DM), chronic ischemic heart diseases, allergic pulmonary diseases, hyperlipidemia, rheumatic disease, allergic otolaryngologic diseases, and allergic dermatological diseases. To ensure that the systemic disease has a sufficient interval to affect the corneal status, we only chose the systemic disease with an interval longer than two years as the parameters in the current study.

### 2.5. Statistical Analysis

SAS version 9.4 (SAS Institute Inc., Cary, NC, USA) was employed for the statistical analyses in our study. The descriptive analysis was employed to present the distributions of demographic data, systemic morbidities, and the numbers of PK in our study group. Then, we administered the Cox proportional hazard regression to produce the adjusted hazard ratios (aHRs) and corresponding 95% confidence intervals (CIs) of each demographic data and systemic diseases for the infectious keratitis development. The effect of other demographic data (including age) and systemic diseases were adjusted in the Cox proportional hazard regression while analyzing the effect of specific covariates. In the subgroup analyses, all the participants were divided based on the difference of age (older than 60 years or not) and sex (male or female). After that, Cox proportional hazard regression was employed again to evaluate the influence of DM and chronic ischemic heart disease on the infectious keratitis development in each subgroup. The statistical significance was regarded as *p* < 0.05 in our study, and a *p*-value lower than 0.0001 was displayed as *p* < 0.0001.

## 3. Results

### 3.1. Baseline Characteristics

**Figure 1:**
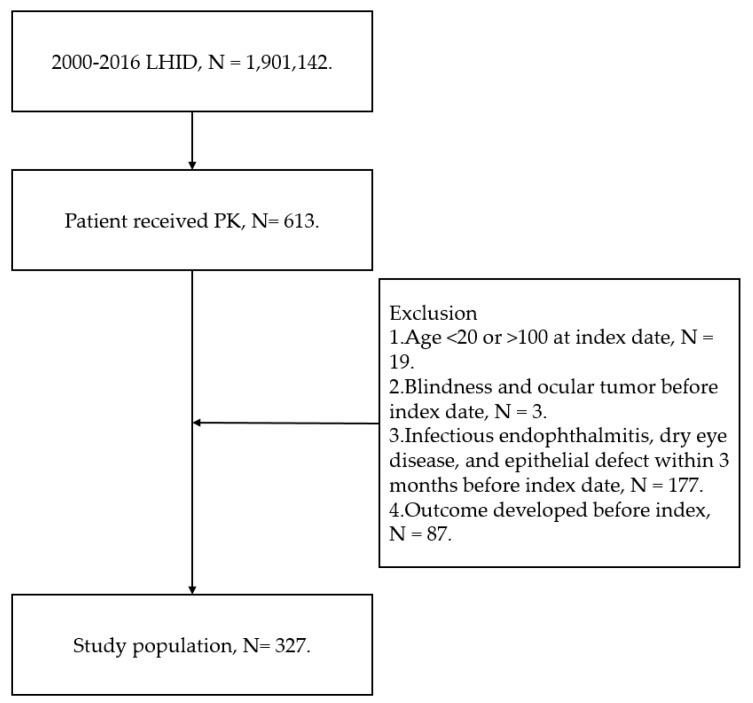
The flowchart of patient selection. PK: penetrating keratoplasty; *N*: number.

A total of 327 participants that received PK were enrolled, and the flowchart of patient selection is available in Figure 1. The basic characteristics of our study population are presented in the Table 1. There were 183 males and 144 females in our study, and the age interval with the highest patient numbers was 70 to 79 years old. The most prevalent systemic disease in our study group was hypertension, which occurred in 110 patients, while rheumatic disease only developed in 5 patients (Table 1).

### 3.2. Risk Factors for Infectious Keratitis

There were 68 patients who developed infectious keratitis after the whole follow-up period of 16 years (Table 2). When comparing the effect of demographic data and systemic diseases on the development of infectious keratitis, DM (aHR: 1.440, 95% CI: 1.122–2.874, *p* = 0.0310) and chronic ischemic heart disease (aHR: 1.534, 95% CI: 1.259–3.464, *p* = 0.0273) demonstrated significant effects on the development of infectious keratitis. In addition, the patients aged 20–39 years old illustrated a significantly lower possibility of infectious keratitis development (aHR: 0.212, 95% CI: 0.052–0.864, *p* = 0.0305). The other parameters did not show significant influence on the occurrence of infectious keratitis (all *p* > 0.05) (Table 3).

In the subgroup analyses, DM revealed a significant influence on the development of infectious keratitis in both subgroups aged younger than 60 years old and older than 60 years old (*p* = 0.0282 and 0.0056, respectively). Nevertheless, the effect of chronic ischemic heart disease on infectious keratitis was only significant on those aged older than 60 years old (*p* = 0.0094). On the other hand, the effects of DM and chronic ischemic heart disease on infectious keratitis development were significant in both sexes (all *p* < 0.05) (Table 4).

## 4. Discussion

Briefly, our study demonstrates the significant effects of DM and chronic ischemic heart disease on infectious keratitis development after PK surgery. Moreover, the effect of DM is significant in all the age and sex subgroups, while the effect of chronic ischemic heart disease is non-significant in patients younger than 60 years old. Additionally, the PK patients aged between 20 and 39 years old illustrated a lesser tendency regarding infectious keratitis development.

Several visual-threatening complications may develop after the arrangement of PK [6]. Infectious endophthalmitis is a rare but dreadful post-PK complication with an incidence of 0.38% [21]. Regarding infectious endophthalmitis occurring in patients receiving PK, the 5-year graft survival rate was 27% and the mean best-corrected visual acuity was only 1.13 LogMAR [22]. Postoperative ocular hypertension and glaucoma is another severe complication of PK, which has a prevalence of up to 60% and is one of the most common etiologies of corneal graft failure [23]. The risk factors for post-PK glaucoma include the preexisting glaucoma, aphakia status, bullous keratopathy, previous failed graft, and pseudophakic status [23]. Infectious keratitis, on the other hand, shows an incidence of about 10% in the patients receiving PK [9], which was higher than the rate of infectious keratitis in the general population [24]. The common microorganism of infectious keratitis after PK included some ocular surface microorganisms such as Gram-positive bacteria, and surgical approaches are not uncommon for the infectious keratitis after PK [8]. The risk factors for infectious keratitis include dry eye disease, corneal lesions, contact lens wear, trauma episodes, and topical steroid application, according to the previous literature [12,13]. In addition, some systemic diseases like DM and systemic immunosuppression had been found to correlate with the development of infectious keratitis in the general population [25,26]. We speculate that since the cornea after PK is weaker than the cornea in its usual condition, some systemic diseases may elevate the rate of infectious keratitis, especially in those receiving PK. This concept is supported by the results of our study, at least partially.

In the present study, the existence of DM and chronic ischemic heart disease correlated to the higher rate of infectious keratitis after PK. To our knowledge, a rare study reported this correlation between the two diseases and post-PK infectious keratitis. Moreover, the effects of several risk factors like age and sex were adjusted in the multivariable analyses, and the ocular risk factors, including dry eye disease and several corneal diseases, were excluded in the present study; thus, the interference of ocular surface conditions in our analysis can be decreased at least to some degree. Consequently, both DM and chronic ischemic heart disease may be an independent risk factor for the infectious keratitis development after PK. The treatments for PK in our population include topical antimicrobial agents, topical steroids, and systemic steroids according to the ATC codes, which are routine treatments for post-PK status; thus, our finding may be applicable to the general post-PK patients. The presence of DM was associated with several systemic disorders and corneal lesions in previous studies [27,28]. In a previous study, the presence of DM could impair the corneal epithelial healing process and corneal endothelial function [29,30], and the poor-controlled DM correlated to a worse therapeutic outcome of ocular surface surgery [30]. Additionally, the structure of the cornea would be damaged in the patients with DM regarding the corneal hysteresis, corneal resistance factor, and central corneal thickness [31,32], and DM also contributes to the higher incidence of corneal graft failure, which is a prominent risk factor of post-corneal transplantation infectious keratitis [16,33]. In addition, DM also correlated to other infections, like granulomatous amoebic encephalitis, which was observed in both the murine model and the real-world patient [20,34]. In clinical practice, we also found that the DM patients had a higher incidence and severity of infectious keratitis. Due to the above evidence, it is possible that the negative effect of DM would cause a higher rate of infectious keratitis in those who received PK. On the other hand, there is no literature reporting the association between chronic ischemic heart disease and infectious keratitis [35]. However, chronic ischemic heart disease is associated with the lipid deposits in the cornea [36,37,38], and the presence of chronic ischemic heart disease could elevate the possibility of systemic infection [19]. Thus, chronic ischemic heart disease may also increase the risk of infectious keratitis after PK. In the physiological aspect, the existence of both DM and chronic ischemic heart disease is associated with a high level of c-reactive protein [39,40], and the c-reactive protein expression would also elevate in those with infectious keratitis [41]. The above evidence further illustrates that our findings could be real rather than incidental.

Concerning the subgroup analyses, DM owned the universal influence of infectious keratitis development in all age groups, while the presence of chronic ischemic heart disease only elevated the risk of infectious keratitis in patients older than 60 years. In previous studies, hyperglycemia led to significant impairment on corneal tissue [42], while there was no evidence that hyperlipidemia contributed to similar corneal lesions. We proposed that the influence of DM on infectious keratitis is greater than that of chronic ischemic heart disease, and the effect of chronic ischemic heart disease on infectious keratitis only presents in patients older than 60 who generally have poorer health conditions compared to their younger counterpart. Furthermore, old age had been identified as a risk factor for post-PK infectious keratitis in a previous study [14,17]; thus, a weak indicator like chronic ischemic heart disease may cause a higher risk of post-PK infectious keratitis in the elderly population. Regarding the subgroups stratified by sex, both DM and chronic ischemic heart disease demonstrated significantly the effect of infectious keratitis development in both sexes. The possible explanation for the similar effect of DM and chronic ischemic heart disease in both sexes is that the effects of these two disorders are strong enough to elevate the risk of infectious keratitis evenly in those with known risk factors of infectious keratitis. Additionally, the incidence of infectious keratitis after PK was only marginally higher in the male population in previous research conducted in Taiwan [43]. The aHRs of infectious keratitis were also numerically higher in the male sex compared to the female sex, which is comparable to the previous literature [43].

Regarding other factors that were enrolled in our study, the young age range of 20 to 39 years old is a protective factor for infectious keratitis in the current study. There is no consensus that age younger than 40 years old is a protective factor for infectious keratitis formation. For the possible reason of this finding, we speculated that the patients aged 20 to 39 years old in Taiwan may put more attention on their health condition and the trauma-associated infectious keratitis due to agricultural products and foreign bodies, often aged around 45–55 years old based on a previous article [44]. The other demographic and systemic disease factors did not exhibit significant influence on the development of infectious keratitis after PK surgery. The different age and sex did not present a predilection on infectious keratitis development [12], and our results correspond to previous experience.

There are still some limitations of the present study. First, the case numbers of our research are relatively insufficient compared to other population-based studies and may cause some statistical bias. Additionally, due to the nature of claimed-database research, some crucial factors, including the severity of systemic diseases, the blood sugar control in the population with DM, the results of laboratory data, the external eye photography of the cornea after PK, the exact microorganism that induced infectious keratitis, the treatment outcome after infectious keratitis, and the recurrence of infectious keratitis if it existed, cannot be investigated. Also, this study is a case–control design, and the level of evidence was lower than those with prospective randomized control trial or cohort study. We did not evaluate the incidence of infectious keratitis in agricultural workers with specific diseases like DM or hypertension, which would decrease the precision of our results. Finally, we excluded the patients with dry eye disease and epithelial defects previously, which may reduce the external validity of this study since the two diseases are not-uncommon in those who received PK surgery.

## 5. Conclusions

In conclusion, the presence of DM and chronic ischemic heart disease is correlated with a higher risk of developing infectious keratitis after adjusting multiple parameters. Furthermore, the effect of DM on infectious keratitis after PK is more universal, while chronic ischemic heart disease only influenced patients older than 60 years old. Consequently, additional care for those with these two morbidities and a schedule for PK surgery may be recommended. Further, large-scale prospective trials to evaluate the influence of DM and chronic ischemic heart disease severity on infectious keratitis development after PK surgery are mandatory.

## Figures and Tables

**Table 1:** The initial characteristics of the study population.

Characters	Number of Patients Who Received PK (*N* = 327)
Index year	
2001–2003	85 (25.99%)
2004–2006	53 (16.21%)
2007–2009	54 (16.51%)
2010–2012	56 (17.13%)
2013–2016	79 (24.16%)
Sex	
Male	183 (55.96%)
Female	144 (44.04%)
Age	
20–39	46 (14.07%)
40–49	38 (11.62%)
50–59	62 (18.96%)
60–69	74 (22.63%)
70–79	77 (23.55%)
≥80	30 (9.17%)
Urbanization	
Urban	195 (59.63%)
Sub-urban	94 (28.75%)
Rural	38 (11.62%)
Systemic disease	
Hypertension	110 (33.64%)
DM	62 (18.96%)
Chronic ischemic heart diseases	32 (9.79%)
Hyperlipidemia	57 (17.43%)
Cerebrovascular disease	29 (8.87%)
Allergic pulmonary diseases	35 (10.70%)
Rheumatic disease	5 (1.53%)
Allergic otolaryngologic diseases	35 (10.70%)
Allergic dermatological diseases	73 (22.32%)

PK: penetrating keratoplasty, *N*: number, DM: diabetes mellitus.

**Table 2:** Incidence risk of study event.

Outcome	Patients Who Received PK (*N* = 327)
Follow-up person months	25,131
Infectious keratitis	68 (20.80%)
Non-infectious keratitis	259 (79.20%)

PK: penetrating keratoplasty, *N*: number.

**Table 3:** Adjusted hazard ratios of each covariate for infectious keratitis.

Parameters	aHR	95% CI	*p*-Value
Sex			
Male	Reference		
Female	1.354	0.777–2.359	0.2850
Age			
20–39	0.212	0.052–0.864	0.0305 *
40–49	Reference		
50–59	0.716	0.288–1.780	0.4722
60–69	0.913	0.349–2.386	0.8524
70–79	0.791	0.297–2.107	0.6385
≥80	0.414	0.097–1.764	0.2331
Urbanization			
Urban	Reference		
Sub-urban	1.027	0.533–1.978	0.9374
Rural	1.144	0.456–2.867	0.7745
Systemic disease			
Hypertension	1.224	0.678–2.210	0.5016
DM	1.440	1.122–2.874	0.0310 *
Chronic ischemic heart diseases	1.534	1.259–3.464	0.0273 *
Hyperlipidemia	0.668	0.301–1.482	0.3211
Cerebrovascular disease	0.570	0.200–1.624	0.2927
Allergic pulmonary diseases	1.130	0.444–2.876	0.7972
Rheumatic disease	0.693	0.083–5.785	0.7352
Allergic otolaryngologic diseases	0.967	0.345–2.712	0.9494
Allergic dermatological diseases	1.061	0.581–1.935	0.8477

aHR: adjusted hazard ratio; CI: confidence interval; DM: diabetes mellitus. * denotes a significant correlation with the development of infectious keratitis.

**Table 4:** The effects of diabetes mellitus and chronic ischemic heart disease on infectious keratitis stratified by age and sex.

Subgroup	aHR	95% CI	*p*-Value
Age < 60 years			
DM	1.382	1.004–2.966	0.0282 *
Chronic ischemic heart disease	0.880	0.597–1.296	0.5163
Age > 60 years			
DM	1.690	1.458–3.039	0.0056 *
Chronic ischemic heart disease	1.856	1.403–5.881	0.0094 *
Male			
DM	1.876	1.240–3.421	0.0404 *
Chronic ischemic heart disease	1.919	1.376–4.465	0.0223 *
Female			
DM	1.278	1.015–2.460	0.0434 *
Chronic ischemic heart disease	1.141	1.144–2.119	0.0318 *

aHR: adjusted hazard ratio; CI: confidence interval; DM: diabetes mellitus. * denotes a significant correlation with the development of infectious keratitis.

## Data Availability

Due to the policy of the National Health Insurance Administration in Taiwan, the raw data of this study are not available.
